# Attention

**Published:** 1877-09

**Authors:** 


### ATTENTION.

We ask of the readers of the Register a careful examination and study of the article, in the present No. "The Involintary Action of the Nervous System, by Dr. J. J. Caldwell, M. D., of Baltimore. This is an exceedingly interesting and instructive subject, one that none can study without profit.

Dr. C—— is eminently qualified to treat this subject, having under very favorable circumstances, given many years to its study, having had hospital practice, in both civil and military service for eight years, and a large private practice for over twenty years. During all this time he has given special attention and study to the nervous system in both its normal and abnormal phases; so that his knowledge and experience in this direction cannot be otherwise regarded, than with special consideration and regard.

